# Supplementary material for: Identification of photoperiod-induced specific miRNAs in the adrenal glands of Sunite sheep (Ovis aries)
Source: Front Vet Sci. 2022 Jul 22;9:888207. doi: 10.3389/fvets.2022.888207 (PMC9354845; doi:10.3389/fvets.2022.888207)
Supplement: Supplementary file 1 [file Data_Sheet_1.ZIP › Supplementary file/miRDeep2 file.zip!/miRDeep2 file/Know_Structure/SP42A3/oar-mir-380.pdf]

miRBase precursor : oar-mir-380
Total read count : 1486
oar-miR-380-5p read count : 19
oar-miR-380-3p read count : 1467
remaining reads : 0

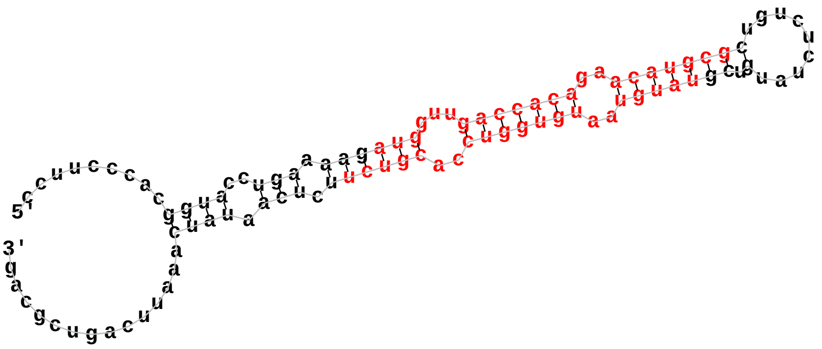

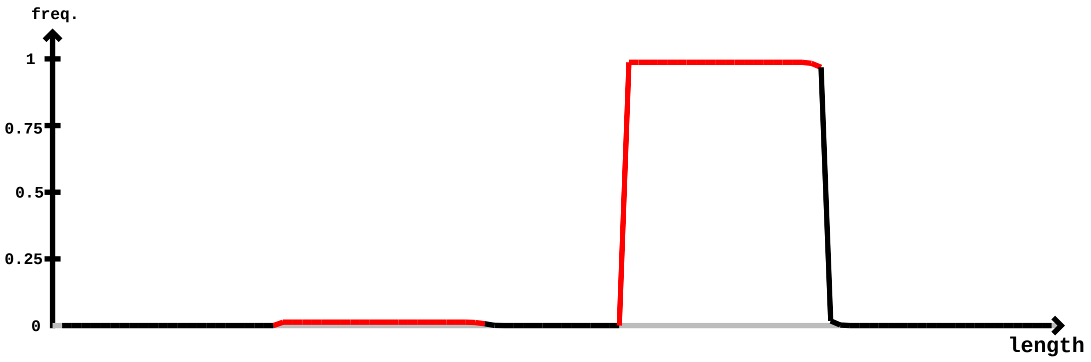


```
                                   oar-miR-380-5p
                                                             oar-miR-380-3p
5'- ccuucccacgguaccugaaaagaugguugaccacagaacaugcgcugucucuaugucguauguaaugugguccacgucuucucaauaucaaauucagucgcag   -3'    exp
    .........((((..(((.((((((...(((((((..((((((((.........).)))))))..))))))).)))))).))).)))).............     reads    mm    sample
    ......................augguugaccacagaacaug.............................................................     2        0     seq
    ......................augguugaccacagaacaugc............................................................     7        0     seq
    ......................augguugaccacagaacaugcg...........................................................     9        0     seq
    ......................augguugaccacagaacaugcgc..........................................................     1        0     seq
    ..........................................................uauguaaugugguccacgu..........................     6        0     seq
    ..........................................................uauguaaugugguccacguc.........................     22       0     seq
    ..........................................................uauguaaugugguccacgucu........................     1413     0     seq
    ..........................................................uauguaaugugguccacgucuu.......................     23       0     seq
    ..........................................................uauguaaugugguccacgucuuc......................     3        0     seq
```
